# Is dengue a threat to the blood supply?

**DOI:** 10.1111/j.1365-3148.2009.00916.x

**Published:** 2009-04

**Authors:** D Teo, L C Ng, S Lam

**Affiliations:** * Blood Services Group, Health Sciences AuthoritySingapore; † Environmental Health Institute, National Environment AgencySingapore

**Keywords:** blood supply, dengue fever, dengue virus

## Abstract

Dengue is the most common arthropod-borne infection worldwide, affecting at least 50 million people every year and endemic in more than 100 countries. The dengue virus is a single-stranded RNA virus with four major serotypes. Infection with one serotype confers homotypic immunity but not heterologous immunity, and secondary infection with another serotype may lead to more severe disease. The major route of transmission occurs through the *Aedes aegypti* mosquito vector, but dengue has also been transmitted through blood transfusion and organ transplantation. Infection results in a spectrum of clinical illness ranging from asymptomatic infection, undifferentiated fever, dengue fever, dengue haemorrhagic fever (DHF) to dengue shock syndrome (DSS). Dengue is spreading rapidly to new areas and with increasing frequency of major outbreaks. A trend has also been observed towards increasing age among infected patients. This will impact blood supply availability as more blood donors are deferred because of dengue infection or exposure to infection. The risk of transmission through transfusion of blood from asymptomatic viraemic donors will also increase. Although screening tests for dengue and effective pathogen reduction processes are now available for the blood supply, the value of implementing these costly measures needs to be carefully considered. Demand for platelets and fresh frozen plasma will rise with increasing number of DHF/DSS. Evidence-based guidelines for the clinical use of these blood components in the management of patients with DHF/DSS have not been well established, and inappropriate use will contribute to the challenges faced by blood services.

The World Health Organization classifies dengue as a major international public health concern. Some 2·5 billion people or two-fifths of the world’s population are at risk of infection. Dengue haemorrhagic fever (DHF) is endemic in more than 100 countries in Africa, the Americas, Eastern Mediterranean, South-East Asia and the Western Pacific regions (World Health Organization, 2008).

Worldwide, it is estimated that 50 million dengue infections occur every year with 500 000 requiring hospitalization; 2·5% of those affected may die (World Health Organization, 2008). These figures are likely to be underestimated as the numbers reported are influenced by different surveillance and reporting systems as well as varying interpretations of case definitions and the presence of missed and silent infections (Guha-Sapir & Schimmer, 2005).

## EPIDEMIOLOGY

Prior to the 1940s, dengue was characterized by epidemics that occurred relatively infrequently, and generally, each location involved a single viral serotype. When sufficient numbers of vectors and susceptible hosts for the particular serotype were reached, a sudden sharp rise in transmission occurred. In contrast, the predominant pattern of global transmission today is hyperendemic dengue, where there is continuous circulation of multiple viral serotypes within a constant pool of susceptible hosts and vectors.

The first known epidemic of DHF in Asia occurred in Manila, Philippines in 1953–1954, to be followed by Bangkok, Thailand in 1958 and Malaysia, Singapore and Vietnam in the 1960s. With the economic boom and associated urbanization, it rapidly spread to involve most of Asia. By mid-1970s, dengue had overtaken malaria to become the leading cause of hospitalization and death among children in the region. Increased spread is also seen into suburban and rural areas (Gubler, 2002).

In the Americas, with the eradication of *A. aegypti* vector to control yellow fever, only sporadic cases were reported until the early 1980s when a large outbreak occurring in Cuba marked the start of epidemic spread to the Pacific and the American tropics. Dengue has been prevalent in tropical Africa, although DHF is rare. It occurred occasionally in the temperate regions of North Africa and the Mediterranean regions until the 1990s when epidemics were reported in the Comoros. Dengue continues to expand its territory, and the only continents that currently do not experience dengue transmission are Europe and Antarctica.

The factors that have contributed to the dramatic expansion of dengue include population growth, urbanization, inadequate water management, lack of effective mosquito control and convenient global travel. In the last few years, changes in weather patterns may have contributed to the expansion in the habitat range of the mosquito vector. Increased epidemic activity may also increase the rate of viral genetic change and emergence of strains or genotypes with greater epidemic potential (Gubler, 1998; Messer *et al.*, 2003; Dash *et al.*, 2006).

Although DHF is primarily an illness of children, surveillance studies in recent years in several countries have reported increasing age among infected patients (Liu *et al.*, 2003; Gupta *et al.*, 2006; Khan *et al.*, 2007; Ooi *et al.*, 2006). In Singapore, this has been ascribed to lowered herd immunity in the population as a result of reduced dengue in the 1970s and 1980s. Studies have shown that *A. aegypti* bites adults more frequently than children (De Benedictis *et al.*, 2003), and together, these could explain the higher incidence rate among young adults in Singapore.

## DENGUE VIRUS AND THE MOSQUITO VECTOR

The dengue virus is a single-stranded RNA virus belonging to the Flaviviridae family. The viral genome is approximately 11 kb in length and is surrounded by an icosahedral nucleocapsid covered by a lipid envelope. The mature virion has three structural (core, membrane-associated and envelope) and seven non-structural (NS1, NS2a, NS2b, NS3, NS4a, NS4b and NS5) proteins.

The envelope protein is involved in the main biological functions and is responsible for binding and transport into host cells. It is also associated with the induction of neutralizing antibodies and development of protective immune response in the host. The non-structural proteins are expressed as both membrane-associated and secreted forms and have been implicated in the pathogenesis of severe disease (Gubler, 1998).

There are four serotypes classified according to their immunological properties – DEN-1, DEN-2, DEN-3 and DEN-4. Infection with one dengue serotype confers lifelong immunity against that serotype but only transient protection against infection by other serotypes. All four serotypes have been associated with outbreaks, having seen DEN-2 as the predominant serotype of primary infection during the outbreak in 2005 (Savage *et al.*, 1998; Messer *et al.*, 2003; Gupta *et al.*, 2006; Ooi *et al.*, 2006). Recent data have also demonstrated that the various genotypes within each serotype possess varying epidemic potential (Anderson & Rico-Hesse, 2006).

The primary vector for the dengue virus is the *A. aegypti* mosquito, although the virus may be transmitted by the *Aedes albopictus* and *Aedes polynesiensis* as well. Infected humans are the main carriers and amplification host of the dengue virus. Female mosquitoes acquire the virus by biting infected humans in the viraemic phase and become infective after an extrinsic incubation period of 7–14 days. Subsequently, the mosquito may transmit the virus during every feeding. The length of the extrinsic incubation period is dependent on the ambient temperature and the virus involved, both of which affect the replication rate of the virus in the vector.

*Aedes aegypti* is a highly domesticated mosquito that breeds in artificial containers such as water storage tanks, subterranean pits, flowerpot trays and other ornamental containers. The vector is known to prefer to rest indoor, although studies have shown that they may seek oviposition outdoors. Peak biting activity is at dawn and dusk. The multiple feeding behaviour of *A. aegypti* and its preference for human hosts are believed to contribute to the explosive spread of dengue virus, even in the presence of a low *A. aegypti* population (Scott *et al.*, 1997).

## LABORATORY DIAGNOSIS

Laboratory diagnosis of dengue can be performed by viral isolation, serological tests, dengue antigen tests and molecular detection.

Virus isolation for dengue is performed by inoculation of the sample into live mice, live mosquitoes or cell cultures. Successful detection of virus may be affected by the presence of interfering antibodies and the heat-labile nature of the virus. It is normally used as a confirmatory test, being impractical for diagnosis or screening on a large scale.

Dengue-specific immunoglobulin M (IgM) and immunoglobulin G (IgG) enzyme-linked immunosorbent assay (ELISA) is widely used in diagnosis as it is relatively inexpensive, has good sensitivity and is quick and easy to perform. IgM is detectable at 3–5 days after infection, peaks at about 2 weeks and declines to undetectable levels over 2–3 months. IgG becomes elevated after 9–10 days and persists at detectable levels for life. During secondary infection, IgG increases rapidly to much higher levels within 1–2 days after infection. Because the virus shares antigenic epitopes with other flaviviruses, the presence of cross-reactive antibodies may interfere with serological diagnosis (World Health Organization, 1997).

The dengue NS1 antigen-capture ELISA is useful for detection of dengue early in the disease. The test sensitivity has been demonstrated to be significantly higher in primary dengue infection (97·3%) than in secondary dengue infection (70·0%), with a positive predictive value of 100% and negative predictive value of 97·3% (Kumarasamy *et al.*, 2007). Although it is useful in the first week of disease and provides evidence of presence of the virus, its effectiveness in screening blood donors has not been established yet.

Like the NS1 test, the dengue RNA test detects viral material that is typically present in the first 5 days of disease. The advantages of the test are good sensitivity and specificity, and the ability to rapidly detect minute quantities of dengue virus material in serum. The disadvantages are the relatively high cost and expertise needed, particularly proper quality control to avoid false positives.

Real-time reverse transcription–polymerase chain reaction (RT-PCR), using either a universal dengue oligonucleotide primer pair or a combination of the four serotype-specific oligonucleotide primers, is widely used for clinical diagnosis and public health surveillance. It has established detection limits of 0·1 plaque-forming units (PFU) mL^−1^ for DEN-1 and DEN-2, 1 PFU mL^−1^ for DEN-3 and 0·01 PFU mL^−1^ for DEN-4 and 88% correlation with virus isolation (Lai *et al.*, 2006).

Group-specific one-step PCR using universal dengue oligonucleotide primer pairs and SYBR green I is widely used for population surveillance as it is fast and cost-effective for mass screening, with a detection limit of 10–4·1 PFU mL^−1^ (Shu *et al.*, 2003; Lai *et al.*, 2006).

A prototype dengue RNA transcription-mediated amplification (TMA) assay (Gen-Probe, Inc., San Diego, CA, USA) was developed for use in large-scale screening of blood donor samples and uses target genomic sequences that are highly conserved across all four serotypes. The analytical sensitivity of the assay has been established at a detection of 14·9 copies mL^−1^ at 95% detection limit and 3·5 copies mL^−1^ at 50% detection limit for DEN-1, with comparable sensitivity for all four serotypes, and with a specificity of 99·91% (Linnen *et al.*, 2008).

## CLINICAL FEATURES

Dengue infection can produce a spectrum of clinical illness – undifferentiated fever, dengue fever (DF), DHF and dengue shock syndrome (DSS). In infants and children younger than 15 years, the patient is usually either asymptomatic or has a mild undifferentiated febrile illness with maculopapular rash.

DF is characterized by the sudden onset of high fever, severe headache (especially in the retro-orbital area), arthralgia, myalgia, nausea, vomiting and rash. Infants and younger children tend to present with an undifferentiated febrile disease, often with rash. The acute febrile illness lasts approximately 2–7 days.

DHF is clinically defined by high fever, haemorrhagic manifestations, thrombocytopenia and evidence of plasma leakage (Table 1). Four grades of severity have been defined (Table 2), where grades III and IV are considered to be DSS. The presence of thrombocytopenia with concurrent haemoconcentration differentiates grades I and II DHF from DF (World Health Organization, 1997). Clinical deterioration usually occurs towards the end of the febrile phase when the patient progresses to the phase of plasma leakage.

**Table 2:** World Health Organization grading severity of DHF*

Grade I	Fever accompanied by nonspecific constitutional symptoms; the only haemorrhagic manifestation is a positive tourniquet test and/or easy bruising
Grade II	Spontaneous bleeding in addition to the manifestations of grade I patients, usually in the forms of skin or other haemorrhages
Grade III	Circulatory failure manifested by a rapid, weak pulse and narrowing of pulse pressure or hypotension, with the presence of cold, clammy skin and restlessness
Grade IV	Profound shock with undetectable blood pressure or pulse

* From World Health Organization (1997).

**Table 1:** World Health Organization case definition for DHF and DSS*

**Case definition for DHF**
The following must be present:
Fever or history of acute fever lasting 2–7 days, occasionally biphasic
Haemorrhagic tendencies, evidenced by at least one of the following:
A positive tourniquet test
Petechiae, ecchymoses or purpura
Bleeding from the mucosa, gastrointestinal tract, injection sites or other locations
Haematemesis or melaena
Thrombocytopenia (100 000 cells mm^−3^ or less)
Evidence of plasma leakage because of increased vascular permeability, manifested by at least one of the following:
A rise in the haematocrit equal to or greater than 20% above average for age, sex and population
A drop in the haematocrit following volume replacement treatment equal to or greater than 20% of baseline
Signs of plasma leakage such as pleural effusion, ascites and hypoproteinaemia
**Case definition for DSS**
All the above four criteria for DHF must be present plus evidence of circulatory failure manifested by:
Rapid and weak pulse
Narrow pulse pressure [<20 mmHg (2·7 kPa)]
Or manifested by:
Hypotension for age
Cold, clammy skin and restlessness

* From World Health Organization (1997).

Plasma leakage manifests as tachycardia, hypotension, pleural effusions and ascites develop. Bleeding from the gastrointestinal tract or epistaxis may occur, with menorrhagia in females. Epigastric discomfort, myalgia, vomiting, diarrhoea and abdominal pain are common in adults. Tender hepatomegaly is observed in all patients and splenomegaly in some. Shock, plasma leakage and marked thrombocytopenia are more common in children, whereas internal haemorrhage is more frequent as age increases (Hammond *et al.*, 2005).

In DHF, the decline in platelet counts tends to precede plasma leakage, and platelet counts and packed cell volume are often used to monitor for impending deterioration (Tai *et al.*, 1999). Haemoconcentration may be difficult to ascertain in areas where a significant proportion of the population carries the thalassaemia genetic trait or where there is a high degree of iron deficiency (Khan *et al.*, 2007).

DSS is a medical emergency associated with very high mortality. Severe plasma leakage results in prolonged shock, accompanied by metabolic acidosis, which in turn precipitates disseminated intravenous coagulation (DIC). Massive haemorrhage or encephalopathy may develop, the former requiring intensive blood transfusion support.

Severe dengue infections can result in complications such as liver failure, DIC, encephalopathy, myocarditis, acute renal failure and haemolytic-uraemic syndrome. These are generally rare and usually seen in patients with DHF grades III and IV (Malavige, 2006).

There are no specific drugs against the dengue virus, and management is symptomatic and supportive. DF is typically a self-limiting disease with a mortality rate of less than 1%, although the convalescent phase may sometimes be prolonged in adults. Successful management of DHF lies in proper management of fluid balance. Early identification of the plasma leakage phase with prompt resuscitation has been shown to reduce complications and improve outcome. When adequately treated, the mortality rate for DHF can be as low as 1%, but if untreated, it can exceed 20% (World Health Organization, 1997, 2008).

## PATHOPHYSIOLOGY

The primary pathophysiology seen in DHF is an acute increase in vascular permeability that leads to leakage of plasma into the extravascular compartment, resulting in haemoconcentration and decreased blood pressure. Increased vascular permeability is shown to result from endothelial gaps in the peripheral vascular bed. No necrotic or inflammatory lesions are seen, suggesting that the changes are functional and likely to be caused by a short-acting mediator.

The principal mechanisms by which dengue virus infection causes DHF are still not clearly elucidated and are believed to be a result of viral and host factors that involve the activation of T cells, production of cytokines/chemokines and disturbance of the haemostatic system. These result in the release of other cytokines that mediate the systemic effects of plasma leakage and circulatory insufficiency. In addition, there is evidence for increased apoptosis and endothelial cell dysfunction (Gubler, 1998; Guglani & Kabra, 2005).

More severe infection is observed to occur in secondary infection than in primary infection (Pancharoen *et al.*, 2001). Various theories have been put forward to explain this observation, including the phenomenon of antibody-dependent enhancement and more recently the theory of original antigenic sin (Mongkolsapaya *et al.*, 2003).

During the early stage of infection, thrombocytopenia occurs as a result of bone marrow hypocellularity resulting from direct dengue infection of haematopoietic progenitor cells and stromal cells (Nakao *et al.*, 1989). As the fever settles, bone marrow hypercellularity is seen, and increased destruction from immune-mediated clearance of platelets becomes the primary mechanism for thrombocytopenia (Mitrakul *et al.*, 1977).

## RISK FACTORS FOR DHF

There are age-related differences in susceptibility to DHF/DSS. Although children infected with dengue virus tend to manifest milder clinical symptoms than adults, they have higher risk of developing DHF/DSS (Hammond *et al.*, 2005; Ooi *et al.*, 2006). It is postulated that children have a greater propensity for vascular leakage under normal physiological conditions than adults, and this results in less ability to accommodate additional factors that increase vascular permeability.

Hospitalization and death rates for severe and very severe dengue are highest in those younger than 15 years and those older than 60 years (Guzmán *et al.*, 2002).

Elderly patients older than 65 years are more likely to develop severe illness, with higher risk of hospitalization and death when infected with dengue virus than youths and younger adults (García-Rivera & Rigau-Pérez, 2003). Age above 65 years, history of dengue infection, diabetes mellitus, hypertension and renal insufficiency have been identified as being significantly associated with DHF/DSS (Lee *et al.*, 2006).

Genetic influences, including human leucocyte antigen allele associations, may play a part in disease susceptibility and severity (Wagenaar *et al.*, 2004). In Haiti, very few DHF cases are recorded despite a transmission rate of 30% and evidence showing the presence of all four serotypes in the population (Halstead *et al.*, 2001). There is similar paucity of DHF cases in Africa despite the presence of dengue virus in the population, suggesting the presence of a dengue resistance gene.

## VIRAEMIA AND TRANSMISSION

The primary mode of transmission for the dengue virus occurs through the mosquito vector. After the bite of an infected mosquito, the dengue virus enters the body and replicates within the cells of the mononuclear phagocyte lineage. There is usually an incubation period of 3–14 days (average 4–7 days). The period of viraemia roughly corresponds to the period of fever, peaking at the time of or shortly after onset of illness and remaining detectable for various periods (typically 2–7 days). There is no correlation between the duration of viraemia and serotype. Some studies have reported correlation between viraemia and disease severity, whereas others report no correlation (Gubler *et al.*, 1981; Vaughn *et al.*, 1997; Murgue *et al.*, 2000).

Infectivity during the incubation period has not been well defined, partly because of the lack of an animal model for dengue. In studies involving human volunteers during the 1920s, clinically apparent DF resulted in 80 and 25% of biting experiments at 1 and 2 days before the onset of fever, indicating that infected persons could transmit virus as early as or even earlier than 2 days before symptoms develop (Nishiura & Halstead, 2007).

Vertical transmission has been reported, with intrapartum transmission in at least two cases where the onset of neonatal illness developed on the first day of life (Sirinavin *et al.*, 2004) and one case involving a mother who was diagnosed with DHF just prior to delivery (Rigau-Pérez *et al.*, 2001).

Nosocomial transmission through needlestick injury and mucocutaneous exposure has also been reported. The case of mucocutaneous transmission involved a healthcare worker who developed dengue infection after blood from a febrile-dengue-infected traveller splashed onto her face (Chen & Wilson, 2005).

Transmission of dengue infection has been reported from donor to recipient in one case of living donor renal transplant (Tan *et al.*, 2005). The clinical presentation and course of illness was similar to that of an immunocompetent patient, except for prolonged course of illness (19 days) and duration of thrombocytopenia (12 days).

Transmission during a bone marrow transplant was reported in one instance during a dengue epidemic in Puerto Rico in 1994 (Rigau-Pérez *et al.*, 2001). The bone marrow recipient developed fever 4 days after the transplant and subsequently died. The donor developed fever and headache 2 days after the marrow was harvested.

There are only two reported instances of transmission through blood transfusion. The first involved a patient in Hong Kong who developed fever 3 days after a blood transfusion, associated with moderate neutropenia, severe thrombocytopenia and hypotension responsive to fluid therapy. The donor was asymptomatic at the time of donation but developed mild symptoms of DF 1 day after blood donation. An archived sample from the donation also tested positive for dengue virus by RT-PCR (Chuang *et al.*, 2008; C. K. Lin, Hong Kong Red Cross Blood Transfusion Service, Hong Kong, pers. comm.).

The second involved the transmission of dengue from an asymptomatic blood donor who developed an acute febrile illness the day after donating blood. Look back investigation confirmed dengue infection in the recipients of the three blood products from his donation. Two recipients had DF with some evidence of capillary leakage, whereas the platelet recipient had asymptomatic seroconversion. All recovered without sequelae. A stored serum sample from the donation tested positive for DEN-2 by RT-PCR (Tambyah *et al.*, 2008).

The small number of reports of transfusion transmission could be because of the fact that it is difficult to differentiate between non-mosquito transmission and mosquito-borne infection in endemic areas where the vector is widespread. Many infections may also result in mild or asymptomatic illness that is not recognized as transfusion-acquired infection, and diagnostic laboratories to document infections and their sources are often not available in many endemic countries (Chen & Wilson, 2005).

## ASYMPTOMATIC INFECTION

Various studies have shown the presence of asymptomatic or subclinical infection, which can range from 0·77 to 87% depending on the population studied (Ooi *et al.*, 2006). It is estimated that for every one symptomatic case, there can be 6·7 cases that are asymptomatic (Chen & Wilson, 2005). There is no correlation with total dengue incidence, hospitalization rates, age or sex, although one study found silent transmission to occur more commonly in the 15- to 40-year-old age group.

In a study of 329 healthy volunteers in a province in Thailand with high rate of dengue infection, 29 (8·8%) had a serum sample positive for dengue IgM, of which two samples tested positive for viral RNA (Poblap *et al.*, 2006). Cluster sampling studies around index cases in Indonesia detected eight post-enrolment asymptomatic dengue infections of 785 volunteers over a 2-year period, of which two demonstrated viraemia by RT-PCR (Beckett *et al.*, 2005). Virus was isolated in 215 of 3189 (6·7%) persons in a study evaluating the dynamics of transmission of dengue virus in a dengue epidemic area of Colombia; most of whom were asymptomatic (Méndez *et al.*, 2006).

Two recent studies reported viraemia in blood donations collected from four countries experiencing high dengue transmission rates. In the first study, 12 (0·07%) of 16 521 blood donations collected in Puerto Rico tested positive using the dengue-specific TMA nucleic acid amplification test (NAT) (Mohammed *et al.*, 2008). Supplemental testing using RT-PCR was positive in four samples (viral loads 2 × 10^3^ to 8 × 10^7^ copies mL^−1^), and live virus was recovered from three of the PCR-positive samples.

In the second study, samples from asymptomatic blood donors in Honduras, Brazil and Australia were obtained during periods of clinical dengue outbreaks and screened using the dengue-specific TMA assay (Linnen *et al.*, 2008). Nine (0·30%) of 2994 Honduran samples tested positive, of which 8 were confirmed by RT-PCR (viral loads 3 × 10^4^ to 4·2 × 10^4^ copies mL^−1^) and 4 samples yielded infectious viruses. Three (0·06%) of 4858 Brazilian samples tested positive, of which 2 were RT-PCR positive (viral loads 12 and 294 copies mL^−1^); viral isolation was not performed for these samples.

## IMPACT ON BLOOD DONORS

Dengue is spreading rapidly into new areas, and major outbreaks are occurring with greater frequency. More than half the world is now endemic for dengue, and there is increasing likelihood that blood donors in these countries will be infected or exposed to infection. As the numbers continue to increase, the effect on blood donor attendance will reach levels sufficient enough to impact significantly on the blood supply.

In most countries, donors who have been infected with dengue virus are deferred for periods of up to 6 months (Table 3) or even longer if they have received blood transfusions. Blood donors who donate regularly will be particularly affected, including apheresis donors who may donate as often as 12 or 13 times a year. Healthy uninfected donors whose family members or working colleagues are infected with dengue are also likely to be deferred from donating blood. These donors would also have less time available to donate blood, as they would need to care for sick family members or take on increased work responsibilities as a result of more workplace illness.

**Table 3:** Dengue and donor deferral

Country	Donor deferral measures for dengue
Singapore*	6 months deferral for history of dengue infection
	3 weeks deferral for history of fever
	No travel-related deferral for dengue
Hong Kong*	6 months deferral for history of dengue infection
	2 weeks deferral for history of fever
	No travel-related deferral for dengue
Sri Lanka*	No specific deferral for history of dengue infection
	2 weeks deferral for history of fever
	No travel-related deferral for dengue
Australia†	4 weeks deferral for history of dengue infection
	No travel-related deferral for dengue
New Zealand‡	4 weeks deferral for history of dengue infection
	No travel-related deferral for dengue
UK‡	2 weeks deferral for history of dengue infection
	No travel-related deferral for dengue
United States‡	4 weeks deferral for history of dengue infection
	No travel-related deferral for dengue

* Endemic for dengue.

† Non-endemic except parts of Northern Australia.

‡ Non-endemic.

In the past, dengue affected mainly children who do not donate blood. As this trend reverses and the modal age of infection increases, dengue will increasingly affect the segment of population that do donate blood – youths and young adults – further reducing blood donor availability. The annual incidence of dengue infection in Singapore was 180·6 per 100 000 population in 2007, with highest incidence in the segment of population older than 15 years. It is estimated that dengue infection has reduced the blood donor pool by 0·2%; this excludes donors deferred for symptoms related to dengue and exposure to dengue, which is estimated to lose a further 1·1% of donors in 2007.

## RISK OF TRANSFUSION-TRANSMITTED DENGUE

Increased prevalence in the population increases the risk that blood will be collected from a viraemic donor during the asymptomatic or subclinical phase of infection. Transmission of dengue through blood collected from asymptomatic donors has been demonstrated in the two reports from Hong Kong and Singapore, both occurring during the height of epidemics in these countries (Chuang *et al.*, 2008; Tambyah *et al.*, 2008). Blood services in countries experiencing dengue epidemics will need to decide whether stronger measures are needed to protect the blood supply.

During infectious disease outbreaks such as dengue, severe acute respiratory syndrome (SARS) and chikungunya disease, most countries have adopted risk reduction strategies that focus on excluding donors who may be at higher risk of infection or who may be exhibiting early symptoms of infection. In the case of dengue, this has relied mainly on questioning the donor for a recent history of travel to outbreak areas and for symptoms of fever, rash or malaise.

The relatively low sensitivity and specificity of blood safety measures based on donor history has long been debated. Travel-related deferrals in particular lead to high rates of unnecessary donor deferral. In addition, there is often a negative effect on the donor of being deferred, and many donors do not return even if the deferral is temporary (Halperin *et al.*, 1998). Introducing such measures during outbreaks of dengue is likely to exacerbate the problems of dwindling donor attendance and decreasing blood collection described earlier. For example, in Singapore, the SARS outbreak in 2003 and chikungunya outbreak in 2008 resulted in donor deferral rates of 4 and 3%, respectively.

In a population where dengue is endemic, exclusion on the basis of symptoms also fails to address the risk of collecting blood from asymptomatic viraemic donors. Studies so far suggest that there is at least a 1–2 days period of infectivity prior to the development of symptoms. The presence of dengue viraemia in asymptomatic persons, including blood donors, is well documented during dengue epidemics.

The efficiency of transmission depends on a combination of factors: amount and stability of virus, volume of viraemic blood transfused and immune status of the recipient. Although dengue viraemia titres in vertebrate hosts are usually in the range of 10^5^–10^9^ copies mL^−1^ (Chen & Wilson, 2005), it is likely that lower levels and shorter duration of viraemia occur during asymptomatic infection compared with DF or DHF. Mohammed *et al.* (2008) and Linnen *et al.* (2008) demonstrated the presence of viral loads of at least 2 × 10^3^ to 8 × 10^7^ copies mL^−1^ and 12 to 4·2 × 10^4^ copies mL^−1^, respectively, in healthy blood donors.

Experience with West Nile Virus (WNV) suggests that not all positive donations are infectious. In these two studies, live virus was recovered from 7 of 21 positive donations, indicating that these donations were capable of transmitting infection to recipients. The viral loads present were in the order of 4·4 × 10^2^ copies mL^−1^ to 8·12 × 10^7^ copies mL^−1^. In contrast, cell cultures were negative in five donations with viral loads between 78 and 7·4 × 10^3^ copies mL^−1^. Although this suggests that lower viral loads are associated with lower risk of infectivity, WNV has been transmitted from transfusions with estimated viral loads as low as 0·06 PFU mL^−1^ or 24 copies mL^−1^. It is therefore possible that larger volumes involved during blood transfusion could still transmit infection despite lower viral load concentrations.

The risk of infection will also depend on the size of population exposed to the infection and immunity because of prior infection. Infection may not occur if the majority of individuals have previously been infected and developed protective antibodies.

Studies on the prototype dengue NAT system showed sufficient sensitivity and early detection to enable interdiction of infective donations. However, it is likely that individual sample NAT rather than mini-pool NAT will be required for dengue screening because of the relatively low levels of dengue RNA (Mohammed *et al.*, 2008). At the time of writing, at least two dengue NAT systems for blood supply screening are in development from Gen-Probe Ltd. and Roche Diagnostics, Ltd., and it is possible that these will be commercially available within the next few years. While the cost of such systems is not available as yet, it is unlikely that they will be less than the current WNV-NAT or hepatitis B virus NAT (HBV-NAT).

An alternative strategy to testing is pathogen reduction, which has the added advantage that it is effective against other infections that can be transmitted through blood, such as the chikungunya virus and WNV. Preliminary studies using the amotosalen-HCl and long-wavelength ultraviolet light (UVA) system on apheresis platelets have shown effectiveness against the dengue virus (Lam *et al.*, 2007). Pathogen reduction technology is not yet available for red cells, and this has somewhat limited its impact. Nonetheless, as more vector-transmitted infections emerge to threaten the blood supply, pathogen reduction of platelets and plasma coupled with selective red cell inventory hold may be useful in reducing transmission risks without unnecessary donor deferral.

Regardless of whether a new screening assay or pathogen reduction method is selected, the recent experience with human immunodeficiency virus NAT (HIV-NAT) and hepatitis C virus NAT (HCV-NAT) suggests that this will involve high costs and expensive technical expertise (Marshall *et al.*, 2004). There is little doubt that transfusion-transmitted infections will represent only a small fraction of total infections during an epidemic. In countries with high dengue prevalence, the introduction of a screening test or pathogen reduction process is therefore likely to prevent infections through blood transfusion, but the proportion would be low in comparison to the total number of infections (Table 4).

**Table 4:** Options for minimizing dengue risk in the blood supply

Strategies	Endemic countries	Non-endemic countries
No specific measures taken for dengue	Risk of transfusion-transmitted dengue increased, dependent on prevalence in donor population and proportion of donors with asymptomatic infection	Risk of transfusion-transmitted dengue low, dependent on proportion of donor population who may recently be exposed to dengue infection through travel
	No direct cost to blood service, but indirect cost from patient morbidity from transfusion-transmitted infection and loss of confidence in blood supply safety	No direct cost to blood service, but indirect cost from loss of confidence in blood supply safety in event of a transfusion-transmitted infection occurring
Donor qualification – deferral of at-risk donors, e.g. symptoms of fever, travel history, exposure to dengue patients, etc.	Deferral based on exposure not feasible when disease is endemic, unable to exclude early and asymptomatic infection	Deferral based on exposure feasible, able to reduce risk of accepting donations from early and asymptomatic infected donors
	Nonspecific, leads to high donor loss	Low donor loss, dependent on proportion of donor population likely to travel to endemic countries
	Low cost-effectiveness	Cost-effective
NAT testing of donations for dengue	Able to detect asymptomatic infection	Able to detect asymptomatic infection
	Donor loss dependent on specificity of test system	Donor loss dependent on specificity of test system
	Expensive	Expensive
	Cost-effectiveness depends on prevalence of asymptomatic infected donors	Low cost-effectiveness
Pathogen reduction	Able to reduce transmission risks	Able to reduce transmission risks
	Expensive and only available for platelets and plasma currently. May result in reduced product yields	Expensive and only available for platelets and plasma currently. May result in reduced product yields
	Low cost-effectiveness for dengue alone	Low cost-effectiveness for dengue alone
	Increased cost-effectiveness depends on ability to reduce risks of other transfusion-transmitted diseases as well	Increased cost-effectiveness depends on ability to reduce risks of other transfusion-transmitted diseases as well

Dengue carries a high economic burden on society, in terms of medical costs and control measures, as well as reduced workforce productivity (Gubler, 2002; Guha-Sapir & Schimmer, 2005). In developing countries with limited resources, the decision to implement a costly risk reduction strategy into the blood supply must take into consideration its cost-effectiveness in reducing disease morbidity in relation to the overall health situation in the country.

Cost-effectiveness of risk reduction strategies for transfusion-transmitted diseases depends on the clinical consequences on recipient health, and whether infection acquired through blood transfusion results in more serious disease. There are few studies reported on the clinical effects of superimposed dengue infection on patients. A retrospective analysis in Brazil of primary dengue infection in 27 renal transplant recipients showed similar clinical picture and outcome as in the general population (Azevedo *et al.*, 2007). A report from French Guiana of 22 pregnant women who had DF during pregnancy found no abnormality in their infants but observed a higher foetal death rate than usual, suggesting that dengue infection might increase the risk for foetal mortality (Carles *et al.*, 1999).

Superimposed dengue may subject the patient to additional risk. Tan *et al.* (2005) noted in their case report that the occurrence of DHF early postoperatively posed potential danger to the transplant patient as a result of persistent haemorrhage from cut tissue surfaces from the associated bleeding diathesis. Most recipients of blood transfusion are in the older age group and will therefore be at increased risk of DHF and complications following dengue infection. The higher risks to patients involving secondary infection with a different serotype have also not been studied.

Case studies suggest that dengue acquired through blood transfusion will not result in more severe illness than that acquired through the usual vector routes of transmission. The four patients who acquired infection through blood transfusion in Hong Kong and Singapore experienced relatively benign course of illness and uneventful recovery with no serious sequelae. This could be because of the lower viral loads present in the blood donations collected. Nevertheless, more data will be needed to make a firm conclusion.

## DENGUE AND PLATELET TRANSFUSIONS

Blood transfusion is only indicated in cases where there is significant clinical bleeding. The exact platelet counts at which platelet transfusions should be given in dengue infection are still not well defined, although most clinical guidelines recommend that platelet transfusions are given to patients who develop serious haemorrhagic manifestations or have very low platelet counts – platelet counts falling below 10–20 × 10^9^ L^−1^ without haemorrhage or 50 × 10^9^ L^−1^ with bleeding or haemorrhage.

In children, there is little correlation between platelet count and bleeding manifestations or between platelet count and disease severity (Malavige, 2006). A study of risk factors for haemorrhage in 114 paediatric patients with DSS showed no correlation between bleeding and platelet count, and prolonged duration of shock was in fact the strongest risk factor for haemorrhage (Lum *et al.*, 2002).

In adults, a platelet count of 5 × 10^9^ L^−1^ and packed cell volume >50 are significantly associated with the presence of bleeding manifestations (Malavige, 2006). However, in uncomplicated dengue patients, one study of 120 patients found no difference in morbidity between those with platelet counts above or below 50 × 10^9^ L^−1^ with or without fever (Lye *et al.*, 2008).

Another study of 245 dengue patients showed no correlation between clinical bleeding and platelet count, and 81 non-bleeding patients had counts of less than 20 × 10^9^ L^−1^ (Chaudhary *et al.*, 2006). In contrast, another study of 225 dengue patients suggested that bleeding occurred more often in patients with platelet counts below 20 × 10^9^ L^−1^ (Makroo *et al.*, 2007).

In a study of 106 paediatric patients with DSS with thrombocytopenia and coagulopathy, there was no significant difference in haemorrhage between patients who received preventive transfusions compared with those who did not. Patients who received transfusion had higher frequency of development of pulmonary oedema and increased length of hospitalization (Lum *et al.*, 2003).

Corticosteroids have been shown to be no more effective than placebo or no treatment for reducing the number of deaths, the need for blood transfusion or the number of serious complications (Panpanich *et al.*, 2006). Randomized controlled study of two treatment groups treated with or without intravenous immunoglobulin also showed no effect in hastening recovery of platelet counts in patients with secondary dengue infection (Dimaano *et al.*, 2007).

In contrast, interim data from two randomized placebo-controlled trials in 47 patients with DHF and thrombocytopenia have shown increase in platelet count with anti-D immune globulin (De Castro *et al.*, 2007). The use of recombinant activated factor VII (rFVIIa) may also be useful in DHF with massive bleeding. Effective response was reported in 8/15 (53%) Thai children with grades III and IV DHF and life-threatening bleeding who were treated with rFVIIa (Chuansumrit *et al.*, 2005).

## IMPACT ON BLOOD SUPPLY

**Fig. 1:**
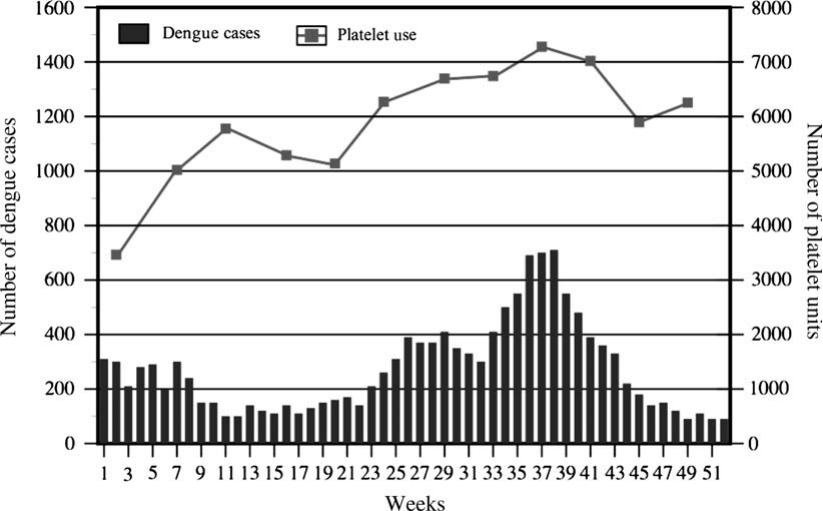
Comparison of platelet usage with number of dengue cases in Singapore in 2005.

Explosive outbreaks of DHF are often associated with an increased need for blood components, especially platelets. When platelets are transfused based on commonly accepted consensus guidelines, about 8% of patients with DF and DHF will require platelet transfusions (Lye *et al.*, 2008). During a large epidemic, this will translate to a significant increase in platelet requirements. During the height of the dengue epidemic in Singapore in 2005, a significant increase in platelet transfusions was recorded (Fig. 1), due to increased requirements from DHF patients.

Inappropriate ordering and use of platelets aggravate these increased requirements and, additionally, expose the patients to unnecessary transfusion risks. Chaudhary *et al.* (2006) report in their study that 35% of patients received unnecessary prophylactic transfusions, and inappropriate doses were given in 89% of transfusion episodes during a DF epidemic. In another study, 31 of 97 patients still received inappropriate transfusions despite the presence of platelet transfusion guidelines (Makroo *et al.*, 2007).

In areas where blood services are less well developed and there is lack of transfusion specialists, lack of knowledge becomes a major factor leading to inappropriate use of blood and blood products. Lum *et al.* (2003) recommend that prevention of haemorrhage in patients with DHF is better achieved through early detection and management of circulatory imbalance and shock than through transfusions. The development of clear and specific guidelines for platelet and fresh frozen plasma transfusion in dengue may be useful in reducing unnecessary use of blood components particularly in areas with limited resources.

Wider access to medical care from health providers with knowledge of DHF can reduce death rates to less than 1% (World Health Organization, 2008). Unfortunately, in many developing countries, rural areas lack such access, and blood supply facilities are often very basic and poorly resourced. As dengue spreads to rural areas, the challenge of providing sufficient supplies of safe blood and blood components to treat DHF/DSS-associated medical emergencies will place a heavy demand on blood services in these countries.
